# Bibliographical Notices

**Published:** 1861-01

**Authors:** 


### BIBLIOGRAPHICAL NOTICES.

A Manual of Auscultation and Percussion: Translated from the French of MM. Barth & Roger, by J. A. Pottenger, M. D., St. Louis. 12 mo. pp. 161. From the Translator.

This is a translation of the *Resume* of the larger work of MM. Barth & Roger, as prepared by themselves for the convenience of students. The merits of the work are too well known to need recapitulation. The Translator has performed his task with scrupulous fidelity, and we take pleasure in commending the "Manual" to all who wish for a concise, perspicuous and practical exposition of the subject.

A Handbook of Hospital Practice, or An Introduction to the Practical Study of Medicine at the Bedside. By Robert D. Lyons, Physician to Jervis St. Hospital, Dublin, &c., &c. Reprint by S. S. & W. Wood, 389 Broadway, N. Y. From the Publishers, 1861.

It does not follow that because a work is brief, that therefore it is superficial. This is a fallacy, which we are happy to say, is gradually disappearing from the minds of bookmakers. The work before us is a capital illustration of the *multum in parvo.* It is a practical companion for Dr. Hartshorne's Syllabus, noticed some little time since.

Adopting Dr. William Farre's classification of diseases, now very generally employed for Registration statistics, the Author proceeds to instruct the student how to examine patients, what to observe, the diagnostic signs, the particular methods applicable to local disorders, post mortem examinations, &c., &c. The task has been accomplished in a manner worthy of high praise. The work contains also an Appendix, including directions for writing prescriptions, a glossary and specimen blanks for recording cases. The student who will thoroughly master the contents of this little book, will be richly rewarded in after years. Nor need even experienced practitioners fear any loss of time by careful reading of its pages. They will find their ideas acquiring definiteness by its perusal, and, practically, cannot fail to derive many valuable suggestions.

---

Contributions to the Anatomy of the Spinal Cord.—By Dr. J. B. Trask, San Francisco, 1860. From the Author.

Extracts from this interesting *brochure* will appear as space permits in subsequent numbers. The author has our sincere thanks.

---

The True Physician.—Dr. John Watson's Anniversary Discourse before the New York Academy of Medicine, Nov. 7, 1860. Published by order of the Academy.

A racy readable production, profusely illustrated with anecdote, and characterized throughout by a high professional tone, worthy of the author and the occasion. We make room for brief extracts :

As in literature and the fine arts, so also in medicine, every enlightened nation has its own peculiarities. Local organizations differ, conventional habits and opinions vary, with varying forms of government. The style of education, and the social rank of medical men, are not the same in all countries. Their modes of promotion to posts of honor, the number and nature of these, are not everywhere alike. The tendency to practical inquiry or abstract speculation, the character and freedom of their discussions, even their artistic skill in writing for the press, partake more or less of the national spirit. Every point in these and other national characteristics has its influence upon the judgment. Peculiarities thus engendered are not always recognized by those moving only in the atmosphere of their own nationality and who never breathe the air of other places; but to the stranger, or returning traveler, they are perceptible enough.

"Be sure you're right at first, then go ahead," is an apothegm bequeathed to us by a man worthy of remembrance, as much for his practical common sense as for his deeds of daring among our western pioneers. The latter part of this injunction is now an accepted Americanism, as common to the habits as to the language of our people; but the clause with which it is coupled is too apt to be forgotten. It is hardly of domestic origin. It points to habitual forethought; to times anterior to the rapid march of modern innovation, and not to those whose custom is to act first, and think afterwards.

The well-known words of Jefferson, "free and equal," have been the theme of many commentators, for approval on the one hand or ridicule on the other. There may have been a time when their influence was trifling; but such is not the case at present. They have long since ceased to apply here simply to political freedom and equality. They are now the enunciation of a sentiment common to our people of every rank and station. This, as it affects our own profession, is most conspicuous in our neophytes, and humbler aspirants after notoriety; but it is also occasionally seen in other quarters.

Cullen, we are told, was on one occasion reprimanded for publishing, and was obliged to retract, certain statements in his work on the Materia Medica which had not the sanction of the Faculty at Edinburgh. The time has never come—it probably never will come—for any medical writer here to desist, at the dictation of any school or association, from giving to the world opinions the most crude, or statements the most unworthy of reliance. This, perhaps, is right. The conflict of errors is their best cure. But the privilege of doing wrong does not imply the expediency of wrong-doing.

When the University of Upsal, at the instigation of Professor Rosen, silenced Linnæus and prohibited him from lecturing on botany, simply for the reason that he was yet an undergraduate, the indignant naturalist drew his sword upon his enemy. For, though young, he was already conscious of his own abilities, and felt that he had been injured by a man inferior to himself. In America, the man of talent has nothing to apprehend from such indignities. But every aspirant to the professorial chair is not a Linnæus, even in America; and it is no strange thing for our professors to become familiar with the department of education assigned to them only after receiving official sanction to take charge of it.

In connection with this disposition to rest satisfied with superficial attainments, and in some measure the result of it, is a tendency on the part of many of our people to frequent change of occupation; and of others to undertake more than they can accomplish. Here every country village can supply its man.

> "Who, in the course of one revolving moon,
> Is statesman, chemist, fiddler, and buffoon."

I might pursue this topic. But with these serious drawbacks it is proper to remark that we are not without our advantages; that in connection with the profession at home we can point to honored names and deserving institutions; that if our schools are not all equal to the best-appointed schools in Europe, the fault is not so much in their style of education as in the low requirements for their degrees; and that such of our youth as are desirous of thorough professional training can find at home the amplest opportunities.

In no other nation are books and periodicals, medical as well as others, so cheap and accessible as among ourselves. In no other is the habit of reading so general. In no other are the rewards of honorable enterprise so open to men of moderate means; and in no other is the energy of the individual so frequently or so variously displayed, or certainly rewarded. Hence it is, that if the profession, as a body, here, have not yet secured the front rank in aggressive science, many among them have thus distinguished themselves, and others are in rapid progress towards these. Hence, too, it is, that though our medical luminaries may not be so numerous as in the Old World, the great mass of men here engaged in the active duties of the profession are better read, and better posted on every question of importance to ourselves than the body of professional men of any other nation. And this remark applies to the practitioner in the rural districts and retired hamlets of the United States as well as to those of our larger cities.

Again, without reference to our medical literature, of which, on the whole, we have no reason to be ashamed; or to the numerous and valuable accessions which have been made to practical medicine and surgery, we have our voluntary associations for mutual improvement, and for the collection and publication of important facts and discoveries; our county and State organizations for supervising the general interests of the profession; and, above all, our National Medical Association, which, in its annual migrations, is uniting us all into one body, awakening public spirit, and inciting to concerted and energetic action the members of the profession in every section of our country. Let us hope for its continued influence and growing usefulness.

Again, the meritorious are not the first to complain of ill success. Speaking of himself, the moralist Johnson tells us that he never knew a man of merit neglected, and that all the accusations against the world for the failure of the deserving are unjust. This may not be universally applicable. But, as observed by one of his recent reviewers, it is certain that those who whine the most deserve the least. The man that cries out, "I am neglected," usually merits the neglect he meets. "I hate a complainer," was another aphorism of this distinguished moralist. Men of Johnson's stamp are, indeed, "superior to fortune, and, as he has said of Shakspeare in his low estate, shake off its incumbrances like dew-drops from the lion's mane."*

Daniel Webster, when asked by a young friend if the legal profession were not already overcrowded, is said to have replied to him, "There is plenty of room up stairs." The intimation is applicable also to our own calling. Of the crowds

* London Quarterly Review, January, 1859.

that are yearly ushered into its portals, only the smallest number ultimately reach its highest honors; but for such as are qualified for these there is yet room.

Finally, the meritorious shun that notoriety which comes from eccentricity of manner, or peculiarity of equipage or costume,—holding obliquity of conduct to be associated with obliquity of judgment. The man of affected, no less than of unaffected eccentricities, is rarely a safe adviser, however brilliant he may be on particular occasions. And the man of sinister motives who assumes these, and whose heart is taken up with his own aggrandisement, is unworthy of acknowledgement as a physician; who, like Hamey, should be "a scholar, without pedantry; a philosopher, without taint of infidelity; learned, without vanity; grave, without moroseness; solemn, without preciseness; pleasant, without levity; regular, without formality; nice, without effeminacy; generous, without prodigality; and religious, without hypocrisy." In a word, his ruling principles, his hopes and aspirations, should be elevated, and of such a nature as to call forth his talents to their full effect. Thus moving, his course is safest as well as best. For at every step he is reaping new installments of his reward, and gathering, as he advances in life, the honors for which he was led to look, and which stimulated him to exertion in his earlier years.

---

*A Practical Treatise on the Ætiology, Pathology, and Treatment of Congenital Malformations of the Rectum and Anus.* By William Bodenhamer, M. D. Illustrated by sixteen plates. New York. Samuel S., and William Wood, p. 360.

The author of this work seems to us to have filled a void in surgical literature, since no treatise on the same subject exists in English or any other language. He appears to have exhausted the subject as far as can be judged, without referring to the original sources from which the materials are drawn, and as regards the history, nature, and treatment of these malformations, to have left nothing to be desired.

While so many ponderous and ill-adjusted treatises superficial upon everything and complete in nothing, are being issued, it is a pleasure to welcome the book to which the practitioner may, in case of need, refer with the certainty of being able to find the information sought for. No Surgeon should be without it.

---

Diseases Peculiar to Women, including Displacements of the Uterus. By Hugh L. Hodge, M. D., Professor of Obstetrics and Diseases of Women and Children in the University of Pennsylvania. *Nullisues addictus jurare in verba magistri.* With original illustrations, pp. 442, Philadelphia: Blanchard & Lea, 1860.

We have only time to announce the publication of this very valuable and interesting work. The reputation of the venerable author, and the importance of the subject will at once commend it to the profession. A professional friend, of distinction in this department, has promised a review for our pages next month. The volume can be found at S. C. Griggs, Lake street.

---

Reports on the Illinois Penitentiary. By the Commissioners for the years 1859–60, pp. 92.

This pamphlet contains much of interest to medical men, and is worthy of attentive perusal. So far as appears from the reports, the State penal institutions are in good order and condition. The mortuary list is remarkably small, which evidences a suitable attention to the hygienic department.

Hez. Williams, M. D., Physician at Alton, reports:

" Sixteen hundred and forty-nine patients have been received at the hospital [in eighteen months], and their symptoms of disease, and surgical and medical treatment placed upon its records. Of this number, twelve have died. This small proportion of mortality, and the general good health of the prisoners, is justly attributable to the careful attention bestowed by the officers of the prison on the preservation of scrupulous cleanliness in all departments, to an abundant supply of wholesome food, and to the maintenance of good discipline; facts of which my opportunities of daily observation in the discharge of my duties as surgeon to the hospital, have made me cognizant."

The report of John R. Casey, M. D., the physician at Joliet, is equally favorable. There were during the year 1859, but nine deaths. During eleven months of 1860, but nine deaths, and of this number, three died of phthisis contracted before entering the Penitentiary.

Whole number of deaths in both branches—*thirty* in two years.

---

*To Business Men.*—Your attention is requested to this Journal as an advantageous advertising medium. Enjoying as it does, the largest circulation of any professional journal in the North-West, and with constant additions to its subscription list—going into almost every town and city of any importance in this section of the country—a notice in its advertising columns will be sure of vastly greater publicity than usually befalls advertisements in most periodicals. Terms as moderate as could reasonably be desired.

| | | | | | |
|---|---|---|---|---|---|
| Half page, one insertion, | $5 00 | Six months, | $20 00 | One year, | $30 00 |
| Whole page, " " | $8 00 | " " | $25 00 | " " | $40 00 |

---

Communications.—All communications for insertion in the Journal, or on business matters connected therewith, may hereafter be directed to the Junior Editor,

DR. J. ADAMS ALLEN,

Box 4458, Chicago, Illinois.

### RUSH MEDICAL COLLEGE.

NINETEENTH ANNUAL SESSION—1861-62.

DANIEL BRAINARD, M. D., President,
*Professor of Surgery and Clinical Surgery.*

JAMES V. Z. BLANEY, M. D.,
*Professor of Chemistry and Pharmacy.*

J. ADAMS ALLEN, M. D.,
*Professor of Principles and Practice of Medicine, and Clinical Medicine.*

JOS. W. FREER, M. D.,
*Professor of Physiology, Microscopical and Surgical Anatomy.*

DE LASKIE MILLER, M. D.,
*Professor of Obstetrics and Diseases of Women and Children.*

EPHRAIM INGALLS, M. D.,
*Professor of Materia Medica and Medical Jurisprudence.*

ROBERT L. REA, M. D., Secretary,
*Professor of Anatomy.*

EDWIN POWELL, M. D., Demonstrator.

The annual announcement will be issued soon.

☞ For information with regard to the institution, address the Secretary, R. L. REA, M. D.
*Chicago, Ill.*

